# Transradial Coronary Angiography and Intervention: Current Concepts and Future Directions

**DOI:** 10.31083/RCM54571

**Published:** 2026-10-16

**Authors:** Gabriela M. Diaz, Edwin A. Takahashi, Ion S. Jovin, Ian C. Gilchrist, Arnold H. Seto, Chayakrit Krittanawong

**Affiliations:** ^1^Department of Internal Medicine, Emory University, Atlanta, GA 30322, USA; ^2^Division of Vascular and Interventional Radiology, Mayo Clinic, Rochester, MN 55905, USA; ^3^Department of Medicine, McGuire Veterans Affairs Medical Center, Richmond, VA 23249, USA; ^4^Section of Cardiovascular Medicine, Heart and Vascular Center, Dartmouth Hitchcock Medical Center, Lebanon, NH 03756, USA; ^5^Department of Medicine, University of California, Irvine, CA 92697, USA; ^6^Penn Highland Healthcare, Monongahela, PA 15063, USA

**Keywords:** radial artery, percutaneous coronary intervention, coronary angiography

## Abstract

Transradial access has become the preferred approach for percutaneous coronary angiography and intervention, largely because this technique reduces vascular complications, bleeding, costs, and patient discomfort compared with transfemoral access. However, anatomical variation, access site difficulties, procedural complications, and other factors can limit success. This review provides a comprehensive overview of the anatomic basis of transradial access, including radial artery anatomy, patient and access site selection, procedural considerations, complications, and emerging applications. This review also highlights key clinical trial data supporting the benefits of transradial access and discusses future directions in technique and technology. Our goal is to provide a practical, comprehensive resource for successful use of this approach.

## 1. Introduction

Transradial access (TRA) and transfemoral access (TFA), the two principal percutaneous coronary access routes, differ significantly in patient selection, cost, and complication rates [1]. Percutaneous TRA for coronary angiography was first described by Dr. Lucien Campeau in 1989, and TRA for percutaneous coronary intervention (PCI) was described by Dr. Ferdinand Kiemeneij in 1992 as an alternative to femoral access to mitigate bleeding complications [2,3]. Over the past 30 years, clinical trial data have demonstrated that TRA is associated with fewer vascular complications, lower costs, and higher patient satisfaction [1,4,5,6]. Reduced bleeding is a consistent advantage, and several studies have shown lower mortality, particularly in patients at high bleeding risk [7,8]. Accordingly, TRA is now recommended by the European Society of Cardiology (ESC), the Society for Cardiovascular Angiography and Interventions (SCAI), and the American Heart Association (AHA) as the preferred access route in acute coronary syndrome (ACS) [7,9,10,11]. In the United States, TRA adoption has increased from 20% of PCI procedures in 2013 to 58% in 2022 [12]. Recently, TRA has also shown favorable results when compared with TFA for chronic total occlusion (CTO) PCI [13]. This review provides an overview of TRA for coronary angiography and PCI, including indications, technique, complications, strategies to optimize patient outcomes, and emerging applications.

## 2. Radial Artery Anatomy

The radial artery arises from the brachial artery distal to the antecubital fossa and courses superficially along the lateral forearm, through the anatomical snuff box, and into the deep palmar arch, where it joins the deep branch of the ulnar artery. Since the hand receives dual arterial supply from the radial and ulnar arteries, collateral blood supply helps protect against limb-threatening ischemia. Compared with the femoral artery, the radial artery is smaller and more prone to spasm, tortuosity, and anatomic variation; however, the size and compressibility of the radial artery also make hemostasis easier and shorten time to ambulation [14]. The radiobrachial region and complex radial loops, observed in 1–2% of patients [15,16], can be difficult to navigate but are usually manageable by an experienced radial operator. Fig. 1 summarizes the anatomical variants to consider.

**Fig. 1.**
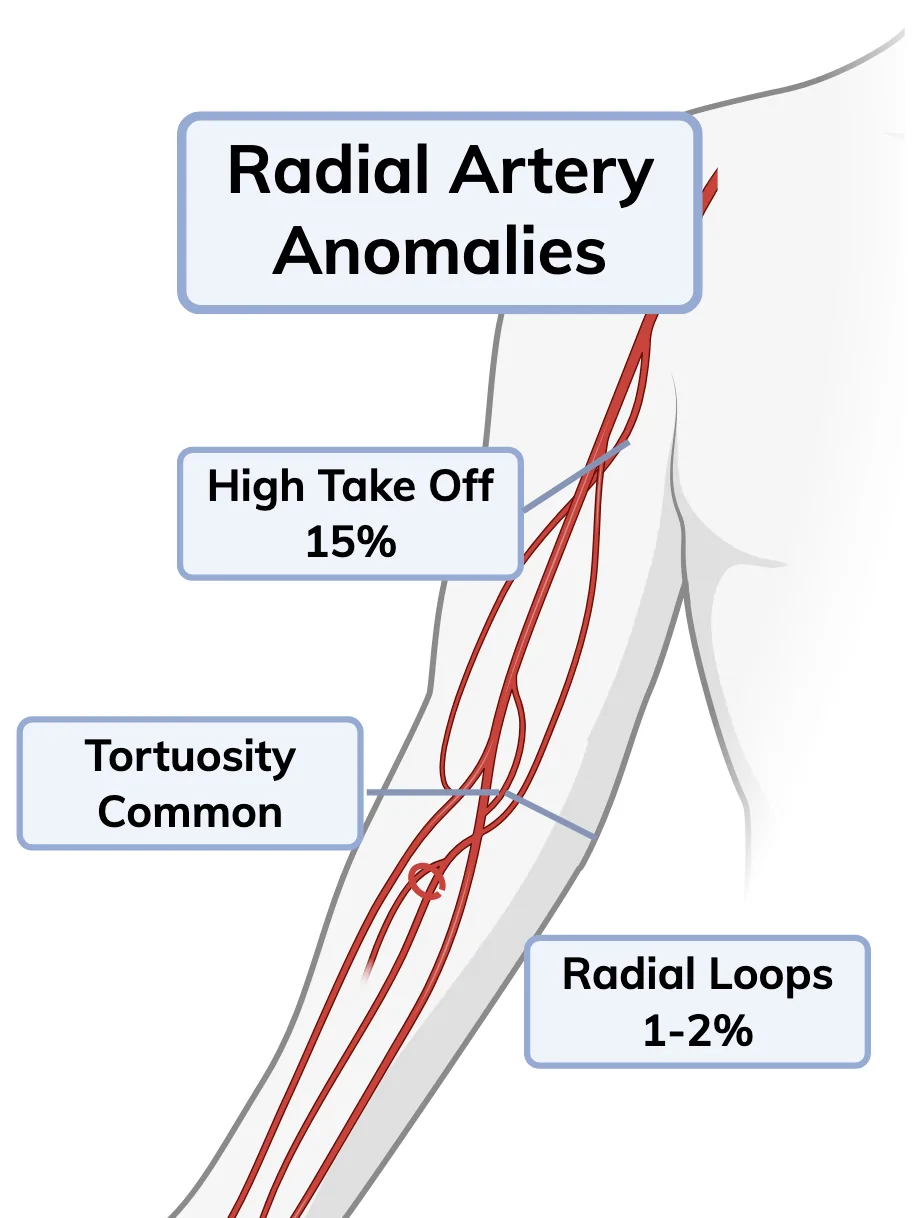
**Radial artery anomalies**.

The radial artery can reliably accommodate up to 6-French (6-Fr) sheaths; larger sheath sizes are associated with radial artery occlusion (RAO). These sheaths may still be used for complex PCI cases, particularly when using sheathless guiding catheters [17,18]. Although the Allen or modified Allen test is commonly used to assess radioulnar collateral hand circulation and exclude patients for TRA, this test has not been shown to provide reliable predictors of hand ischemia complications [19]. Moreover, other important considerations from TRA include preserving the radial artery for future dialysis access in patients with end-stage renal disease [20] and avoiding access in patients with vasospastic or vascular disease, such as peripheral artery disease, Raynaud’s phenomenon, or thromboangiitis obliterans [9]. Ultrasound of the radial artery can also improve procedural planning and first-pass success. In the Radial Artery Access with Ultrasound Trial (RAUST), approximately 700 patients were randomized to palpation-guided or ultrasound-guided TRA, and ultrasound guidance was associated with fewer puncture attempts and a shorter time to access [21]. A recent meta-analysis of 12,621 patients found that the three leading causes of TRA failure or crossover to TFA were failed puncture (35.3%), peripheral occlusion or tortuosity (24.5%), and radial artery spasm (20.1%) [22].

One recommendation for TRA is to use an angiocath needle system with a through-and-through technique to access the vessel. This involves advancing the needle through the anterior and posterior arterial walls and slowly withdrawing the needle until arterial flow is observed; a guidewire can then be advanced through the cannula, which may be easier than direct arterial cannulation [23].

## 3. Case Selection

TRA outperforms TFA across most ACS trial data. Acute Catheterization and Urgent Intervention Triage Strategy (ACUITY) showed less bleeding with TRA (1.9% vs. 4.5%) [24]; Radial versus Femoral Access for Coronary Angiography and Intervention in Patients with ACS (RIVAL) enrolled approximately 7000 patients and found no difference in death or major adverse cardiovascular events (MACE), but fewer vascular complications, with the ST-segment elevation myocardial infarction (STEMI) subgroup showing reduced mortality and ischemic events [25]. Minimizing Adverse Haemorrhagic Events by Transradial Access (MATRIX), a trial with ~8000 patients conducted by experienced radial operators, showed reduced death/myocardial infarction (MI)/stroke at 30 days, without a significant difference by STEMI/non-ST-segment elevation myocardial infarction (NSTEMI) status [26]. Comparison of Early Invasive and Conservative Treatment in Patients with NSTEMI (PRESTO-ACS) and STEMI treated by RADIAL or femoral approach (STEMI-RADIAL) both found less bleeding with TRA, with STEMI-RADIAL also showing fewer vascular complications (1.4% vs. 7.2%) [27,28]. Radial Versus Femoral Randomized Investigation in ST-Elevation Acute Coronary Syndrome (RIFLE-STEACS) noted reduced cardiac mortality, a lower composite endpoint, less bleeding, and shorter length of stay with TRA in STEMI patients [29]. Safety and Efficacy of Femoral Access vs. Radial Access in ST-Segment Elevation Myocardial Infarction (SAFARI-STEMI) found no significant difference in mortality or bleeding, possibly reflecting improved modern TFA closure techniques and exclusion of anticoagulated patients [30]. Meta-analyses across ACS, non-ACS PCI, and other vascular interventions, including an Evaluation of XIENCE Versus Coronary Artery Bypass Surgery for Effectiveness of Left Main Revascularization (EXCEL) left-main PCI subanalysis showing less bleeding with TRA and no difference in death, MI, or stroke, and a 33,311 patient meta-analysis in cardiogenic shock demonstrating lower in-hospital, 30-day, and 1-year mortality, less MACE, and decreased bleeding, further support these findings [9,31]. Overall, the evidence favors a radial-first approach across ACS presentations.

In stable coronary artery disease (CAD), where procedural urgency is less immediate, TRA remains preferred since this approach reduces bleeding and vascular complications, although mortality benefits are less consistent. A meta-analysis of TRA versus TFA in ACS and stable CAD, including 22,843 patients, reported lower all-cause mortality (odds ratio (OR) 0.71; *p* = 0.001), bleeding (OR 0.53; *p* < 0.001), and vascular complications (OR 0.23; *p* < 0.001) [32]. In stable CAD, access choice is more often guided by procedural complexity, the need for large-bore catheters, and operator preference. Even complex PCI can be performed with TRA [13]. In the Femoral or Radial Approach in the Treatment of Coronary Chronic Total Occlusion (FORT CTO) trial, TRA was noninferior to TFA for procedural success (84% vs. 86%; *p* = 0.563) and had fewer access-site complications (2.0% vs. 5.6%; *p* = 0.019) [13]. However, the pivotal trials supporting TRA superiority over TFA predominantly used fluoroscopic or palpation-guided femoral puncture with inconsistent or unreported vascular closure device use, rather than modern ultrasound-guided, closure device-standardized femoral access. Consequently, the magnitude of the advantage of TRA may be inflated by an outdated femoral comparator, and no adequately powered trial has compared TRA with a fully modernized, ultrasound-plus-closure device-standardized TFA protocol at the scale of RIVAL or MATRIX.

## 4. Right vs. Left Radial Access

The choice between right and left TRA depends on right RAO, radial or subclavian tortuosity, radiation exposure, and patient preference for using the nondominant hand. Since most catheterization laboratories are optimized for right TRA, left radial access may require greater arm adduction or even laboratory configuration. Advanced age (particularly ≥70–75 years), female sex, short stature, hypertension, stroke, and prior coronary artery bypass grafting (CABG) are all associated with right subclavian tortuosity and higher TRA failure [33,34,35], with failure rates around 5–10% in these subgroups versus roughly 1–2%. The mechanism is largely anatomic, with older, shorter, and female patients tending toward more tortuous right subclavian and brachiocephalic arteries and shorter ascending aortas, making catheter advancement and guide support harder from the right radial approach.

Another consideration for right or left TRA includes the possible future need for a radial artery conduit for CABG, most commonly the left radial artery, for which TRA may cause trauma to the vessel and preclude its use for CABG [36]. A randomized trial of 1000 patients undergoing diagnostic coronary angiography who were assigned to left or right TRA found no differences in procedural success; however, the trial noted more subclavian tortuosity when accessing the right radial artery (44 vs. 15; *p* < 0.001) and longer fluoroscopy exposure with a right radial approach (3.08 min vs. 2.76 min; *p* = 0.029) [37]. In patients with a prior left internal mammary artery (LIMA) graft, right radial access cannot directly reach the LIMA origin and usually requires an unfavorable retrograde catheter course across the aortic arch; in such cases, left radial access or TFA is preferred since the LIMA takeoff is anatomically much closer to a left-sided approach [38]. Prior CABG is also an independent predictor of TRA failure [39], likely reflecting more diffuse peripheral vascular disease and calcification in this population, which further supports a lower threshold for left radial or TFA in these patients rather than defaulting to standard right radial.

## 5. Radial vs. Distal Radial

Fewer clinical trial data exist that compare conventional TRA with distal TRA, which is accessed through the anatomical “snuffbox” in the dorsal hand space between the thumb and first digit. Distal TRA preserves the proximal radial artery, is associated with lower RAO rates, and can offer easier hemostasis because the vessel is more superficial and lies close to the carpal bones. The distal radial artery in the snuffbox area has an average diameter of 1.5–2.5 mm, which is smaller than the proximal radial artery (2–3 mm). However, standard 6–7 French guiding catheters can be used in most distal TRA, and even larger 8-Fr guiding catheters have been used [40]. Another beneficial aspect of distal TRA is that this approach can mimic femoral ergonomics by positioning the left hand across the body. Unlike standard radial access, a through-and-through approach for arterial puncture is not recommended for distal TRA as this technique can increase pain when the scaphoid and trapezium are contacted [41]. A meta-analysis of 6208 patients undergoing coronary angiography and/or intervention via distal TRA found a lower risk of RAO at follow-up (relative risk (RR) 0.36; *p* < 0.001) compared with standard TRA, but a higher risk of crossover and a longer time to radial artery cannulation. These parameters would likely improve as operators gain more experience with this technique. A Korean registry of 735 patients undergoing coronary angiography or PCI through TRA found that a weak pulse and having performed fewer than 100 distal TRA procedures both predicted procedure failure (odds ratio (OR) 9.994, 95% confidence interval (CI): 7.252–13.774) and (OR 2.187, 95% CI: 1.383–3.456), respectively [42]. Ultrasound use enhances precision and first-pass success. Importantly, across these studies, procedural success rates and major clinical outcomes (death, MI, and stroke) were similar between approaches, which should be balanced against the technical complexity and operator experience.

## 6. Optimal Anticoagulation Strategy

TRA is associated with lower rates of major bleeding than TFA. The optimal anticoagulation strategy for TRA in coronary angiography and PCI aims to prevent RAO and other vascular complications while maintaining procedural safety and reducing recurrent major adverse cardiovascular events. Data have shown that major bleeding after PCI is linked to increased mortality, but we also know that using higher heparin doses decreases RAO incidence [43,44].

Trials comparing unfractionated heparin (UFH) fixed-dosing strategies (2000–3000 vs. 5000 IU) have demonstrated that 5000 IU is superior to <3000 IU for preventing RAO without increasing bleeding risk. A meta-analysis of six randomized controlled trials (RCTs) that comprised around 2000 patients undergoing diagnostic angiography found lower RAO with 5000 IU UFH without significantly increased bleeding risk compared with <3000 IU UFH (4.2% vs. 10.7%, RR 0.40) [45]. Weight-based dosing strategies have shown a similar pattern: in the SPIRIT of ARTEMIS trial, UFH 100 IU/kg significantly reduced the risk of RAO compared with 50 IU/kg for patients undergoing diagnostic transradial coronary angiography (3.0% vs. 8.1%; *p* < 0.001) without BARC type 3 bleeding events; similar rates were noted for BARC type 2 bleeding [46]. The PRACTICAL-HERO trial aimed to compare the effect of high-dose UFH 50 IU/kg, low-dose UFH 25 IU/kg, or placebo (normal saline) on rates of RAO and hematoma [47], thereby providing more nuanced evidence regarding UFH dosing strategies and complication risk.

Bivalirudin remains an option for patients with heparin-induced thrombocytopenia. The MATRIX trial compared bivalirudin to UFH with about half of the patients allocated to TRA and found no difference in MACE at 30 days, regardless of access site [48].

## 7. Wire Selection

Wire selection in TRA should match wire properties to the anatomy and procedural needs of each patient. A standard 0.035-inch J-tip guidewire can be used safely in straightforward anatomy with 5-Fr or larger catheters. Hydrophilic guidewires improve torqueability but carry a higher risk of perforation, especially in smaller vessels. In one randomized trial, a 0.035-inch J-tip standard guidewire had higher technical success than a standard J-tip wire (96% vs. 84%; *p* < 0.001), with failures most commonly due to radial spasm and subclavian tortuosity and no difference in vascular complications [49]. An alternative to a hydrophilic guidewire is a soft, floppy straight-tip (Versacore) wire, which is highly flexible and can be beneficial in high-grade stenoses and multiple lesions [50]. When a hydrophilic wire alone does not resolve resistance, balloon-assisted tracking (BAT), can help by creating a smooth, tapered leading edge with a partially inflated low-pressure coronary balloon; indeed, this approach has shown high success for resolving refractory spasm (~91% in a case series) and reducing femoral crossover, and can be paired with a sheathless guide system when vessel caliber is the limiting factor [51].

## 8. Catheter Selection

Once the wire and catheter reach the aortic root, the diagnostic strategy shifts to selecting a catheter for coronary engagement. TRA provides less guide support than TFA due to its smaller size. The guide catheter maintains stability for advancing procedural devices, but being mindful of catheter-radial artery size differences is key, since RAO occurs more frequently when there is a large discrepancy between catheter and radial artery diameter [52].

A conventional two-catheter method, using separate Judkins left and right catheters, is beneficial for engaging ostia in patients with complex anatomy or left main disease. A one-catheter method, using a single multipurpose catheter (Tiger, Jacky), avoids the added exchange time, contrast, and radiation inherent in radial catheter swaps and is best suited to straightforward diagnostic cases. Data show that using a one-catheter method like the Tiger catheter can reduce procedural times [53,54,55]; however, the Tiger catheter had higher failure rates compared to Judkins catheters in one study [53].

Additionally, in settings where more support is needed, a buddy or anchoring wire can be used. Minimizing catheter exchanges and using a universal catheter are ideal for reducing fluoroscopy time and arterial manipulation; however, care must be maintained to address anatomic variations that may lead to complications with using a universal catheter. Right TRA often requires tailored catheter selection to overcome subclavian and innominate tortuosity. Thus, advising the patient to take a deep breath can help realign the catheter to more easily traverse tortuosity [56].

## 9. Radial Access Complications

### 9.1 Radial Artery Occlusion

RAO is a common structural complication of TRA, occurring in 1–33% of cases [44]. It is important to recognize because it can eliminate future radial access and, rarely, compromise hand perfusion (Table 1). Risk factors for RAO include diabetes, lack of pretreatment with clopidogrel, prolonged occlusive hemostasis, and female sex [9,17,52,57]. RAO can be diagnosed with a post-procedure reverse Barbeau test, where the ulnar artery is compressed, and the waveform is observed; dampening or elimination indicates RAO. Ultrasound is the gold standard for confirming RAO, and absence of antegrade radial flow and/or presence of thrombus would be noted [52].

**Table 1.** **Radial artery complications**.

Complication	Prevention	Solution
Radial artery occlusion (RAO)	Adequate periprocedural anticoagulation; assess radial anatomy with ultrasound; use a micropuncture needle; maintain a sheath-to-artery ratio <1; use a spasmolytic cocktail	Patent hemostasis ± ulnar artery compression for 30–60 min; oral anticoagulation for persistent RAO; urgent vascular evaluation if symptomatic
Radial artery spasm (RAS)	Intra-arterial radial cocktail; hydrophilic-coated sheaths; adequate sedation and analgesia to reduce sympathetic tone; gentle technique	Progressive sedation if persistent; transdermal nitroglycerin paste; sublingual nitroglycerin in refractory cases; warming measures; inflate the blood pressure cuff; angiography to exclude vessel trauma
Radial artery perforation	Avoid excessive force during wire or catheter advancement; use caution with hydrophilic wires in tortuous anatomy	Keep the wire across the perforation to tamponade it; use a long sheath or guide catheter to seal the perforation; apply external compression with a blood pressure cuff; escalate to balloon tamponade, stent placement, or surgical repair if refractory
Compartment syndrome	Avoid excessive anticoagulation or antiplatelet therapy; recognize and manage perforation or hematoma early	Urgent surgical evaluation (fasciotomy) if there is increased pain, tense swelling, or neurological deficits
Radial artery pseudoaneurysm	Avoid multiple access attempts; ensure adequate post-procedure compression; avoid excessive anticoagulation	Compression with a TR Band^TM^; thrombin injection; surgical repair for severe cases
Catheter kinks	Avoid excessive manipulation or over-torquing in tortuous anatomy or spasm	Untwist and insert guidewire → attempt to pull the catheter through an ipsilateral sheath → snare the catheter tip and unwind it → gently inflate the guide catheter with an indeflator → cut the catheter and encase it with the sheath → pull the knot through the skin as a last resort

RAO, radial artery occlusion; TR, transradial.

Preventing RAO with adequate periprocedural anticoagulation is critical, as is minimizing radial trauma by assessing the anatomy of the radial artery with ultrasound, using a micropuncture needle, maintaining sheath-to-artery ratio <1, and a prophylactic or immediate radial spasmolytic cocktail (verapamil 2.5 mg and/or nitroglycerin 0.1–0.4 mg) after vascular access [44,56].

Treating RAO involves early intervention, such as through patent hemostasis, where a pressure band that stops bleeding while allowing forward radial artery flow is applied for several hours. Forced recanalization of the radial artery by compressing the ulnar artery for 30–60 minutes is a helpful adjunct to this method [58]. The PROPHET II trial demonstrated that adding ulnar artery compression to patent hemostasis reduced early RAO from 4.3% to 1% at 24 hours and late RAO at 30 days from 3% to 1% [59]. Oral anticoagulation can also be used for several weeks after the procedure in cases of persistent RAO. Symptomatic RAO is rare but presents with hand pain or ischemic changes and requires urgent vascular evaluation [52,57].

### 9.2 Radial Artery Spasm

Radial artery spasm (RAS) occurs in 4–35% of TRA procedures and is a common cause of prolonged procedure time, procedural failure, and crossover to TFA [60,61]. The etiology of RAS is multifactorial, but any scenario that triggers high sympathetic tone, such as fear, anxiety, or pain, can contribute [62]. Clinical features include resistance to wire or catheter advancement and severe forearm pain. Angiography should be performed to evaluate for spasm and exclude other vessel trauma [60,62]. Risk factors for RAS are similar to those of RAO, including female sex, prior radial procedures, large sheath-to-artery ratio, multiple puncture attempts, and prolonged procedural time [60,61,63,64]. RAS has also been identified as a risk factor for RAO. Prevention and treatment both involve administering an intra-arterial radial cocktail consisting of a calcium channel blocker and/or nitroglycerin, along with heparin. Hypotension is a common adverse effect of vasodilator medications and should be considered on an individual basis [65]. Increasing sedation to reduce sympathetic tone may be necessary if RAS persists [66]. Further adjunctive treatments include transdermal nitroglycerin paste, and sublingual nitroglycerin has been used in refractory cases [67]. In complex, refractory cases, inflating a blood pressure cuff to 20–40 mmHg above the patient’s systolic blood pressure can promote vasodilation by briefly inducing local tissue hypoxia [68].

Hydrophilic-coated sheaths reduce the risk of RAS. A meta-analysis of eight RCTs found that hydrophilic-coated sheaths significantly reduced the risk of RAS and periprocedural pain or discomfort compared with standard non-coated sheaths (RR = 0.38, 95% CI (0.24–0.60) and RR = 0.47, 95% CI (0.37–0.59)). There was no significant difference in the risk of RAO, hematoma, or pseudoaneurysm [63]. This practice has been widely adopted and has largely replaced standard sheaths. Lastly, warming measures and gentle technical adjustments may alleviate RAS and minimize further complications.

### 9.3 Radial Artery Perforation

Radial artery perforation is an uncommon but potentially serious complication of TRA. Risk factors include using a hydrophilic wire, tortuous vessels such as radial loops or severe angulation, and excessive force during wire or catheter advancement. Perforation should be suspected when advancing a wire is difficult, followed by a “give”. Other associated signs include sudden pain, swelling, or a forearm hematoma [9]. Angiography reveals contrast extravasation. The wire should be kept across the perforation, if possible, to promote internal tamponade. A long sheath or guide catheter can also seal the perforation site [60]. If this is insufficient, external compression can be attempted with a blood pressure cuff proximal to the site [56]. If there is ongoing bleeding or large hematoma formation, protamine should be administered to partially reverse procedural heparin. If unsuccessful, escalation to balloon tamponade, stent placement, or surgical repair can be considered [69,70].

### 9.4 Compartment Syndrome

A serious complication of radial artery perforation is compartment syndrome caused by a hematoma. This complication should be suspected following the development of increased pain, tense swelling, and neurological deficits, and requires urgent surgical evaluation. In a large sample of 51,296 transradial cases, compartment syndrome occurred only twice (0.004%) [71]. Predisposing factors are often excessive anticoagulation and antiplatelet therapy [60].

### 9.5 Radial Artery Pseudoaneurysm

Radial artery pseudoaneurysm is a very rare iatrogenic complication that should be suspected when a painless or painful pulsatile swelling occurs at the TRA site. Risk factors include intense anticoagulation, multiple arterial access attempts, and inadequate post-procedure compression. The RIVAL trial reported a 0.2% risk of pseudoaneurysm in the TRA arm [25]. Radial artery pseudoaneurysm is diagnosed by ultrasound, which shows a contained rupture of all three layers of the arterial wall [72]. Traditional management includes compression with a TR band or thrombin injection, while severe cases require surgical repair.

### 9.6 Catheter Kinks

Catheter kinks are caused by excessive manipulation and over-torquing, particularly in settings of tortuous anatomy or radial artery spasm. This should be suspected when the operator experiences loss of torque, dampened arterial pressure, and inability to advance the wire. Kinks can lead to vascular complications and should be managed with an algorithmic approach that begins with examining the entire wire course under fluoroscopy [60]. A series of maneuvers has been recommended to be attempted in succession. This algorithm starts with untwisting and inserting a guidewire, attempting to pull the catheter through an ipsilateral sheath, endeavoring to snare the catheter tip and unwind, gently inflating the guide catheter with an indeflator, cutting the catheter and encasing it with the sheath, and, if all else is unsuccessful, pulling the knot through the skin [9].

## 10. Future Directions

Challenges remain in optimizing outcomes for patients with risk factors for vascular and systemic complications, including tortuous anatomy, high bleeding risk, chronic kidney disease, and peripheral arterial disease. A promising area for future research is risk stratification to guide decisions on access site, equipment, and technical strategies. Integrating objective data prospectively, such as age, sex, and comorbidities, may provide useful information about the risk category for serious complications for each patient. An artificial intelligence (AI)-based clinical decision support tool was utilized in 642 patients who underwent PCI to risk-stratify for contrast-induced nephropathy and guide maximum contrast volumes. Compared with baseline values, use of this tool reduced the incidence of contrast-induced nephropathy from 10% to 2.18% (*p* < 0.0001) [73]. Such tools could be implemented to improve rates of other common procedural complications, such as RAO, RAS, and bleeding, where known risk factors exist and can be proactively addressed using tailored radial cocktails, anticoagulation regimens, or equipment selection. After the procedure, heightened monitoring and follow-up can be instituted based on risk stratification category.

Alternative access sites have been explored to improve procedural outcomes and reduce access-site complications. The ulnar artery has been used successfully for cardiac catheterization and PCI but has not been utilized as extensively as TRA. Ulnar artery access has been shown to carry a lower bleeding risk than TFA and to offer the same early mobilization benefit as TRA; however, the artery has several characteristics that limit mainstream use: deep location, proximity to the ulnar nerve, and technical difficulty. Despite these limitations, several clinical trials have found similar rates of procedural success, bleeding, and vascular complications compared to TRA [74].

We foresee increased adoption of alternate upper extremity access sites to avoid increased bleeding complications associated with TFA and to preserve radial access for future procedures. Data supporting distal TRA may encourage the development of distal TRA-specific equipment that maintains proximal radial access sites. Increased operator experience with these methods is likely to improve PCI outcomes and facilitate both standardized training and the incorporation of specialized techniques to individualize care.

## 11. Conclusion

TRA is now a standard-of-care approach that continues to be refined to improve safety, standardize techniques, and preserve the radial artery for future procedures. A robust understanding of the various approaches, equipment, and complications is essential to optimizing patient outcomes with TRA. We look forward to the development of risk stratification tools to enhance safety and procedural outcomes.
